# Attitudes and Preferences on the Use of Mobile Health Technology and Health Games for Self-Management: Interviews With Older Adults on Anticoagulation Therapy

**DOI:** 10.2196/mhealth.3196

**Published:** 2014-07-23

**Authors:** Jung-Ah Lee, Annie Lu Nguyen, Jill Berg, Alpesh Amin, Mark Bachman, Yuqing Guo, Lorraine Evangelista

**Affiliations:** ^1^University of California, IrvineProgram in Nursing ScienceIrvine, CAUnited States; ^2^University of California, IrvineDepartment of Family Medicine, Division of Geriatric Medicine and GerontologyIrvine, CAUnited States; ^3^University of California, IrvineDepartment of MedicineIrvine, CAUnited States; ^4^University of California, IrvineDepartment of Electrical Engineering & Computer ScienceIrvine, CAUnited States

**Keywords:** anticoagulation therapy, health apps, health games, mobile health technology, self-management

## Abstract

**Background:**

Older adults are at substantial risk for cardiovascular disorders that may require anticoagulation therapy. Those on warfarin therapy report dissatisfaction and reduced quality of life (QOL) resulting from the treatment. Advances in the area of mobile health (mHealth) technology have resulted in the design and development of new patient-centric models for the provision of personalized health care services to improve care delivery. However, there is a paucity of research examining the effectiveness of mHealth tools on knowledge, attitudes, and patient satisfaction with treatment, as well as self-management, adherence to therapy, and QOL in older adults with chronic illness conditions requiring long-term warfarin therapy.

**Objective:**

The objective of the study was to explore the attitudes and preferences of older adults on warfarin therapy regarding the use of mHealth technology and health games to gain skills for self-management.

**Methods:**

We conducted group and individual interviews with patients (60 years or older) on warfarin therapy at two anticoagulation clinics affiliated with an academic medical center. We held 4 group and 2 individual interviews, resulting in 11 patient participants and 2 family caregiver participants. We used structured questions on three topic areas including medication self-management strategies, mHealth technology use, and health games for exercise. We demonstrated some commercial health apps related to medication management, vitamin K content of food, and a videogame for balance exercise. Discussions were audiotaped and transcribed verbatim. Common themes were drawn using content analysis.

**Results:**

The participants reported awareness of the importance of staying on schedule with warfarin therapy. They also acknowledged that negative experiences of friends or family members who were taking warfarin influenced their desire to keep on schedule with warfarin therapy. In addition, the participants expressed that the use of mHealth technology may be helpful for medication management. They also expressed the need for family support in the use of health technology devices. Moreover, the participants discussed concerns and challenges to use health technology and health games, and provided suggestions on ways to make mHealth technology and health games elder-friendly.

**Conclusions:**

These findings indicate that our older adults on warfarin therapy are interested in mHealth technology specific to warfarin medication management and health games. Further research needs to be done to validate these findings. Elder-friendly designs, technology support, and physical safety using mHealth technology may be useful in this population. These findings can be used to inform a larger study to design and test an elder-centered mHealth technology in this target population.

## Introduction

### mHealth Technology and Older Adults

Advances in the area of mobile health (mHealth) technology and improvements in information technology have resulted in the design and development of new patient-centric models for the provision of personalized health care services [1]. Technological innovations in health care can potentially promote healthy aging and reduce disparities by providing more precise and individualized health care services to patients [1]. In addition, mHealth devices and telehealth platforms support disease management for patients who use the Internet and other electronic communication tools to connect with family and friends and communicate with health care providers [2]. Many mHealth apps available in the market were designed for pediatric patients (eg, asthma, diabetes) and adult patients with chronic conditions (eg, diabetes, heart failure) [3]. However, older adults with chronic diseases can also be end-users of such new health technologies. mHealth technology can potentially empower older adults in self-management and can simplify the complex care systems that many older adults with chronic conditions face [1].

Along with the rise of mHealth technology use in chronic disease treatment, health games including virtual reality and interactive gaming have gained increased popularity in the health care industry as a promising approach for the delivery of health education and services [4,5]. They are also being used as tools for enhancing self-care behaviors and for increasing adherence to therapy [4,6,7]. Research has also shown that the use of health games for exercise (eg, Wii) improves physical function such as balance [8-10], and enhances awareness of risk factors and self-management in a variety of patient populations [5,11,12].

### Warfarin Therapy for Anticoagulation

Older adults who are at substantial risk for cardiovascular disorders including atrial fibrillation, valvular disease, venous thromboembolism, and heart failure require long-term oral anticoagulation treatment (ie, warfarin therapy) [13-15]. Warfarin has been used for several decades in a variety of clinical settings in many countries to prevent and treat patients with thromboembolic risk. Although new oral anticoagulants have been recently introduced and approved by the United States Food and Drug Administration, warfarin is still the most common anticoagulant for patients with thromboembolic risk. Wide spread coverage by health insurance plans in the United States to cover the new oral anticoagulants continues to be a challenge.

Despite the proven benefits from warfarin therapy, older adults report dissatisfaction and reduced quality of life (QOL) resulting from the treatment, leading to poor adherence and decreased treatment efficacy [16,17]. Dissatisfaction with treatment has been attributed to a variety of reasons including the need for frequent visits to health care provider clinics to monitor international normalized ratio (INR) [18], lifestyle limitations (eg, restrictions on diet and activities) [19], and fear of potential side effects (eg, bleeding and/or bruising) [20,21].

Such obstacles, as reduced QOL, have prompted the search for alternative strategies (eg, mHealth) to improve attitudes toward warfarin therapy and reduce perceived barriers to ensure that older adults who require anticoagulation therapy are more optimally and consistently treated [22-25]. Moreover, studies showed that the relationship among patient, health care providers, and tailored educational programs in consideration of age differences should be taken into account for a better warfarin management [26,27]. However, there is limited research on the use of mHealth technology to promote adherence to anticoagulation therapy, and also enhance QOL in older adults on warfarin therapy.

The current study reported in this paper is part of a larger study proposing to expand current mHealth infrastructure, and to broaden its impact to older adults requiring warfarin therapy. The overall goals of the parent study are to design and test a theory-based, culturally appropriate, elderly centered mHealth intervention, and to examine patient satisfaction with and feasibility of the intervention. The purpose of the present study was to explore the attitudes and preferences of older adults treated with blood thinners regarding the use of health technology apps and health games to gain skills for self-management. We have used the findings reported here in the development of an mHealth-based intervention for the larger parent study.

## Methods

### Participants

We held a series of group and individual interviews with older adults on warfarin therapy. The patients were eligible if they were 60 years or older, taking warfarin, and speaking English. Exclusion criteria were: (1) diagnosis of irreversible conditions likely to affect 6 month survival or ability to participate in the study, (2) living in a long-term care facility, or (3) cognitively impaired (evidenced by documentation of dementia or delirium in medical records). The level of cognitive impairment of the participants was also evaluated by health care providers (ie, pharmacists who encounter patients on anticoagulation) when referring the participants, and evaluated by the investigators at the time of interviews. In addition, caregivers (age ≥18) of the eligible patients were also included in the discussion if they accompanied the patient participants.

### Procedures

After obtaining approval for the study from the University of California, Irvine Institutional Review Board (IRB), we used passive and active recruitment strategies from October 2012 through March 2013. First, we recruited the participants attending two anticoagulation clinics affiliated in an academic medical center via flyer postings. Second, pharmacists who encountered the patients at the anticoagulation clinics provided study information sheets to the potential participants. Last, investigators obtained the eligible patient names and phone numbers from the pharmacists at the anticoagulation clinics and contacted the potential patients to inform them about the study and recruit them to participate.

Each group session lasted approximately 60-120 minutes and included 2-6 participants. We also conducted individual interviews if only one participant showed on the day of the scheduled group meetings. All of the group or individual interviews were held in conference rooms within the anticoagulation clinics or the hospital. All of the participants were provided with a study information sheet including the purpose of the study, research members’ contact information, study procedures, and potential benefits and risks associated with study participation. The University of California, Irvine IRB, did not require written informed consent. Research members (1 moderator and 1 assisting research staff) conducted all sessions.

Prior to the discussion, the participants were asked to fill out a brief paper-based survey, including questions on demographic information and participants’ experiences with computers, health technology aids, and health related computer/videogames. We provided magnet reading glasses for the participants to wear, or we read survey questions out loud as needed. During the session, the participants discussed strategies and barriers to medication self-management, as well as use of mHealth technology and health games. The questions asked are presented in Table 1. We demonstrated a few commercial health apps, including a medication reminder app and a Vitamin K food content app, as well as a commercial videogame for exercise. Then, we asked the participants to use each one briefly and share their opinion on each mHealth app. The patient participants received US $30 cash each for their participation after the group meetings/interviews were completed. All discussions were audiotape recorded, and the recordings were then transcribed verbatim.

**Table 1:** Interview questions.

Interview questions	
**I. Medication self-management related**	
	1.Have you ever skipped taking your blood thinner?
	2.What are some reasons that you have skipped doses?
	3.What are some strategies that you have used to manage your medications?
	4.How well did those strategies work for you?
**II. Health technology apps (including computers) related**	
	1.How comfortable are you using computers or electronic devices?
	2.What are some of the reasons that you don’t use computers or electronic devices more often? (barriers)
	3.What are some things that would help you feel more comfortable using these devices?
**III. Computer/video health games related**	
	1.What kinds of computer games or videogames have you played before?
	2.What things make videogames or computer games more enjoyable for you? (Do you like games to move around, do you like to play with other people?)
	3.What things make it more difficult for you to enjoy computer games or videogames?
**The commercial apps that were demonstrated**	
	1.Medication monitor app, the app is designed to manage any of person’s pills or medications, taking pills on time with the app, preventing missing taking pills because of so many things to do or bad memory.
	2.Vitamin K app, the app is designed for people who take warfarin so they need to monitor their foods and warfarin interaction. The app provides Vitamin K level in foods. Users can search food by categories including fast foods, fruit and vegetables, meat and fish, dairy and egg, grain and pasta, and more. Vitamin K levels are rated by colors (Black-extremely high in Vitamin K, Red-high, Yellow-moderate, Green-low, White-extremely low).

### Data Analysis

Data analyses were conducted using Atlas.ti 7.0 qualitative data management software (Atlas.ti Scientific Software Development GmbH). The transcripts were analyzed for emergent themes using content analysis based on the principals of grounded theory [28]. Grounded theory allows for an inductive theory-building approach to coding, which does not require a priori theory, but provides a road map through the process of analysis of qualitative data [28]. All transcripts were read and coded by 3 independent raters. The subsequent primary codes were discussed as a team by the 3 raters (including the moderator) and a qualitative research method expert to create consensus codes. There were 3 research team members (2 coders including the moderator, and 1 experienced qualitative methodologist) that reviewed the consensus codes and synthesized them into common themes, including main themes and subthemes. Then, another experienced qualitative nurse researcher independently reviewed the common themes and compared them with all of the transcripts to verify the quality of themes and relevant quotes.

## Results

### Participant Information

There is an appendix that presents the participant demographic information and their experiences with the use of computers, health technology, and health games (see Multimedia Appendix 1). An abbreviated version of this information is shown below in Tables 2 and 3. There were 8 male patients and 3 female patients that participated in the study. A daughter who was a caregiver of a male patient, and a spouse caregiver of a male patient also participated in the discussion. The mean age of the 11 patient participants was 75 years (SD 7.5). The majority (8/11, 73%) completed high school or higher. There were 10/11 participants that reported that they lived with family.

**Table 2:** Participants’ demographic characteristics.

Demographic characteristics		Frequency, N=11
**Age (year)**		
	Mean (SD)	74.9 (7.5)
	Median	75
	Range	61-89
Gender (male)		8
**Ethnicity/race**		
	Caucasian	6
	Hispanic	3
	Asian	2
Education- high school completion or above		8
Living situation- living with family (vs living alone)		10
Currently employed		2
Insured		10
Having a primary care provider		9
**Comorbidities**		
	Stroke	4
	Diabetes	3
	Hypertension	3
	Arthritis	3
	Heart failure	2
	Atrial fibrillation	1
	Lupus	1
Multiple comorbidity (≥1)		5
**Years taking blood thinner**		
	Mean (SD)	5.45 (4.8)
	Median	5
	Range	3 weeks-6 years
Have ever skipped taking blood thinner? (yes)		6

**Table 3:** Participants’ technology related experience.

Computer experience		Frequency, N=11
Have a home computer		6
Use a computer at all including at home, library, community center, other places		6
**Reasons for computer use**		
	Internet and email	6
	Word processing	4
	Scheduling (calendar or reminder)	2
	Managing household finances	0
	Photos or music	0
	Playing games	0
	Other	1
**Frequency of computer use**		
	Never	5
	Once per month or less	0
	2-3 times per month	0
	Every week	0
	Every day	5
Have ever used a smart phone/tablet for health apps such as medication reminders or weight management (yes)		2
**Frequency of health apps use**		
	Once per month or less	2
**Have ever played computer/video games (yes)**		5
	Computer games	3
	Video games (eg, Wii, PlayStation, Xbox, etc)	2
	Games on phones/tablets	1
**Frequency of game playing**		
	Never	10
	Once per month or less	1

### Medication Self-Management

The participants discussed strategies that they used to manage all their medications including blood thinners (eg, warfarin), and reasons for skipping medications. In general, most of the participants reported that they were able to follow the medication regimens as prescribed, and were not prone to skipping medications. However, they described special circumstances that led to skipped medication doses.

### Strategies for Managing Medications

Most of the participants attributed their adherence with their medication regimen to having developed consistent self-monitoring strategies, such as taking blood thinner pills at the same time every day or using tools to help keep track of their regimen. These tools include digital reminders on a cell phone or alarm clock, written reminders on index cards or calendars, and organizational devices such as pill boxes, dishes, jars, and bottles to divide up the correct type and number of pills to be taken in a given time period. There was one participant that further elaborated that he created backup plans, such as keeping an extra dose of medication in the car, in case he forgot to take his pills at home. Some of the participants stated that they had the support of family members and caregivers who helped remind them to take their medication,

My wife, who works for the school system, has her smartphone with her 24 hours a day, and she programmed the time at 5:00pm and at 8:00pm an alert, and if I’m home, she tells me. If I’m not home, she will call me, “Take your medicine”. So my wife actually reminds me every day...so that’s my plan.

Male participant, age 74

Most of the participants also spoke about their awareness of the importance of staying on schedule with their warfarin. For some, learning about the negative experiences of friends or other family members who struggled to remember doses, and consequently had problems with clotting, served to heighten their awareness of the importance of warfarin. There was one participant that shared a story about her daughter-in-law’s experience,

She ended up with some clotting...That knowledge from dealing with my family having it, my daughter-in-law at first was taking it at different interims and then [her doctors] finally realized that she didn’t realize and told us, “no, you have to take it at a specific time”...so I approached it with that knowledge.

Female participant, age 75

For some, having clear instructions from their health providers about why and when to take warfarin was an important component to their adherence. Some of the participants stated that being knowledgeable about blood clots made them realize it was something they did not want, and so taking warfarin was seen as “good” and “preventive”. Some of the participants reported that their doctors further provided them with information about foods high in vitamin K, which helped them to better manage the foods they ate to prevent drug interactions.

### Reasons for Skipping Medications

The participants reported that from time to time, they might skip a dose of medication for a variety of reasons. The most commonly cited reason was that skipping occurred when their daily routine was interrupted or when they were distracted by something. There was one participant that explained, “You have a big break in your normal routine and you’re out of your normal mode and it just slips by” [Male participant, age 74]. Sometimes the participants forgot a dose because they were “busy doing something” so that keeping up with the dose got “lost in the shuffle”. For one participant, being on multiple medications and caring for his grandchildren sometimes meant that,

Once in a while, they run me around and sometimes I forget. I’ll go all day long and I think, I didn’t even take any pills.

Male participant, age 80

For some, skipped doses were a result of being forgetful. This meant that they forgot to take a dose, or forgot whether or not they had already taken a dose. Skipping medications also occurred as a result of doctor’s orders to prepare for surgery. However, the directions received from doctors for skipping medications was sometimes inconsistent and confusing. For instance, one participant stated that in preparation for a surgery, his surgeon directed him to be off warfarin for 5 days, but that another physician directed him to be off for 2 days. He stated,

So I go back to the [surgeon] and tell the nurse and the nurse says, “that would be all right”. So I’m off maybe two days before and two days after.

Male participant, age 80

### Health Technology for Medication Management

The participants were asked about their level of comfort with using computers and various types of technological devices, including mobile phones and tablets. They were also asked to talk about their experience with playing computer or videogames, and to give their thoughts about a variety of health related games and medication management apps on mobile devices, such as mobile phones or tablet computers.

### Participants Without Technological Experience

Those who expressed a lack of experience with computers and other technological devices, such as smart phones and tablets, mostly cited reasons such as lack of interest, patience, knowledge, training, or that it was challenging to learn or a “waste of time”. Some felt that electronic devices were expensive for people on a “limited budget”, and that these devices may be better suited for people with a “bigger salary”. There was one participant that stated that others did not expect her to use or know how to use technology because of her age. She stated,

[My doctor] didn’t ask me [about Skype]. Probably thought I was too old and didn’t know how or something.

Female participant, age 75

### Participants Without Technological Experience for Health

When prompted to comment on their thoughts about technology specifically for health or medication management, the participants with no reported experience in using technology mostly stated that nontechnological methods were “simple” and easy to use. For example, some of the participants stated that although a pill monitor app on a phone or tablet may be useful for some people, a manual pill box was “convenient” and “works well”. There was one participant that was only on one medication, so she was not concerned about her ability to manage her medication, and did not feel that it was necessary to use an app for reminders. Some of the participants talked about the ease of medication refills without the use of technological reminders or alerts because their doctor coordinates with the pharmacy, and they get telephone calls from the pharmacy when they have medications to pick up. However, some of the participants also said that medication management apps might be useful for those who are “forgetful”, are cognitively impaired, or have a difficult time keeping track of appointments.

Most of the participants had positive reactions to apps that help monitor foods with vitamin K, but some expressed that it might be more useful for “someone else” because they felt they already had good knowledge about what foods are high in vitamin K. There was one participant that said,

I wouldn’t be interested in that at all...once you’ve seen the list and you kind of focus on the ones that are high [in vitamin K], you’ve kind of got it. So in my own case, I don’t worry about what I eat...but for other people, maybe they need it.

Male participant, age 73

A few participants expressed interest in using a similar app for themselves. For instance,

That’s very interesting. It would keep you where you’re supposed to be, and what you’re supposed to do by looking the different products up. I like that...because my family says, “You shouldn’t eat that. You should eat more of this”. With this here I can say, “Yep, don’t need. Yep, do need”, you know. That I like very much.

Male participant, age 75

### Participants With Technological Experience

The participants who reported having some level of comfort with technology had experience with a range of devices. This included things such as the use of mobile phones for taking pictures and texting, work experience with computers and touch screens, and regular use of a personal computer. Many of them stated that they used technology to communicate and keep in touch with friends and family. Some of them used the alarm function on their cell phones as medication or appointment reminders. Many of these participants also spoke about their familiarity with technology as being a result of the ubiquitous use of technology by those around them including friends and family.

### Participants With Technological Experience for Health

The participants who had experience using technology had positive reactions to using technologies to manage health conditions and medications. Many of them expressed favorable views of technology for older adults, and also expressed interest in using or learning to use tablets and phone apps for health and medication management purposes. There was one participant that shared,

A medication monitor app might help the people who are on more than one medicine, because even my daughter-in-law, after she went back to work for a couple hours a day, she got back into the old thing of forgetting the warfarin...Where if she had something like this with her, it would have told her it’s time. I think it would be a great asset to a lot of elderly people.

Female participant, age 75

A few participants elaborated further on the appeal of using mHealth technology for health and medication management. They stated that devices such as computer tablets are “small”, “portable”, and easy to carry around and keep by their side.

### Suggestions to Make Technology Elder-Friendly

Because technological devices can be expensive, a few of the participants suggested that being able to rent a tablet from their health provider would be helpful. Many of the participants were concerned about the specifications of devices and suggested that phones and tablets need bigger screens, bigger keyboards, and simple visual illustrations in order to make them more “age-appropriate”. Apps that can help people easily keep track of their diet and vitamin K consumption were viewed as something that can be useful for older adults. There was one participant that also suggested that since many older adults have multiple medications, a medication management app could be useful,

It would be a great way of seeing if you’re being prescribed something that doesn’t go with the other one...It’s hard to keep track of what all you’re on. That was like I went to my doctor after the surgery and showed him what all I was taking. I took them all in and then he had them on his little screen...I do think that it’s a great way to monitor what the patient is having put in their body because, like you said, sometimes it’s very hard for the doctor to look at a piece of paper and keep a list of all these medications and everything.

Female participant, age 75

However, most of the participants suggested that learning to use apps would be challenging for older adults with technology challenges, and that they would need to be provided with external help. For example, someone may need to help the patient enter all of their medications into an app before they are able to use the app for monitoring.

### Health Games for Exercise

In asking the participants about exercise, the interviewers showed the participants a video clip of someone playing a videogame on the Nintendo Wii designed for exercise. They then elicited responses from the participants about their thoughts on using something similar. Some of the participants were familiar with it and said it was something that they would try for themselves, while some did not think it was something they wanted to try. Positive reactions were mainly elicited from those who had played on the Wii with their grandchildren. The participants offered some concerns and suggestions on how to make exercise games more elderly friendly.

### Concerns About Health Games for Exercise

Many of the participants cited the risk of falls as a reason why they did not want to try videogames for exercise. Many of them were worried that they were too “uncoordinated” to play exercise games, and that they would get dizzy and fall. There was one participant that said,

I fall all the time, come running into stuff...It’s the moving back and forth, I would totally lose my balance on that.

Male participant, age 75

Some of the participants expressed that the idea of playing videogames was something that was not appealing to them, stating that they had “no desire” or in the case of one participant, “I can think of other things I should be doing that are much more important” [Male participant, age 80]. Many of the participants stated that walking was their preferred form of exercise.

### Suggestions to Make Health Games Elder-Friendly

A variety of suggestions were made by the participants to make exercise games more appealing to older adults. Making the games multiplayer was a big appeal. Some of the participants said that they had no interest in playing games alone, but that if it were done with other people or in a group, they would be more interested in participating. Some of the participants also suggested that games be developed for people with varying degrees of physical activity levels. For example, one participant suggested that some older adults would need to perform exercises while sitting down. Another participant said of sitting exercises,

I am physically very active. I can do all of this stuff...This isn’t enough for me.

Male participant, age 74

Several of the participants were concerned that standing on the Wii platform could pose as a falling hazard, and suggested that the addition of a support bar or grab bar to hold onto while exercising would be useful.

## Discussion

### Older Adults and mHealth Technology

This study explored the attitudes and perceptions toward mHealth technology and health games in older adults on oral anticoagulation therapy using a qualitative research methodology. The findings suggest that the use of mHealth technology, such as health apps on mobile devices, may be helpful for managing medications since the participants experienced missing doses of medication occasionally. However, the participants also expressed the need for family support in the use of health technology devices. In addition, the participants discussed concerns and challenges to use, and provided suggestions on ways to make mHealth technology and games elder sensitive.

### Medication Self-Management Strategies

Older adults deal with polypharmacy (multiple medications), due to multiple chronic conditions [29]. Factors contributing to medication adherence in older adults include physiological changes with the aging process such as decline in cognitive ability and multiple morbidities, psychosocial profile, health beliefs, patient-health provider communication/relationship, and social support [30]. Individuals develop their own strategies to keep track of their medications and for refills. The strategies that older adults on anticoagulation therapy used in this study included electronic reminders or calendars for refills, pill boxes, or other tools to store medications. Many of the participants reported family support for medication management. The use of mobile-based short message service (SMS) reminders has been shown in studies to improve adherence to medication in chronic disease conditions including acquired immune deficiency syndrome [31], diabetes [32], or polypharmacy [33]. None of the participants in this study reported use of SMS.

Awareness of the importance of taking medication (warfarin) everyday, and the consequences of not maintaining the regimen, seem to enhance adherence to anticoagulation therapy with warfarin. Such awareness was obtained through experiences and anecdotes from friends or family members who had taken warfarin or through good provider-patient communication. At our medical center, anticoagulation specialized pharmacists lead anticoagulation clinics. The patients with warfarin therapy in this study expressed satisfaction with the level of detail of instruction provided by the pharmacists, including which foods have high Vitamin K content, drugs to avoid while on warfarin therapy, the importance of taking blood tests regularly, and adjusting warfarin doses according to INR. For years, the clinical and economic benefits of pharmacy-led anticoagulation management have been demonstrated [34,35].

Most of the participants in this study confirmed the value of using mHealth technology in the management of medications, but commented that it would be more useful for other people, but not themselves. This may be because the mean length of warfarin treatment in this sample is long (5.5 years). The patients in this sample may have adapted and normalized their routine for warfarin management. mHealth technology may be better targeted to patients new to warfarin or to patients who have unstable INR in order to aid the normalization of medication routines.

### Mobile Health Technology

According to the Pew Internet Research report published in June 2012, slightly more than half of all older adults (65 years and older) in the United States used the Internet via computers, cell phones, or tablets [34]. Approximately 56% of older adults responded in the Pew Internet survey that they owned a cell phone [36]. However, the ownership of mobile phones or tablets is low (3%). There were 1 in 3 older adults who used the Internet that reported that they enjoyed social networking (eg, Facebook, LinkedIn) [36]. In this current study, the older adult participants reported similar experiences with computers and mobile devices. In particular, some of the participants raised concerns related to the economic burden for the use of tablets. Some who reported that they did not often use computers expressed little interest for learning to use mHealth technology for managing their medication or for self care. However, many of the participants commented that health apps specifically designed to promote adherence to warfarin therapy would be helpful. They also commented that family support in the use of mHealth technology would be favorable.

The concept and market for mobile technologies in health care have been developing rapidly in recent years [1]. Substantial interest toward mobile technologies in health care is growing alongside the movement toward individualized medicine. The mHealth industry seeks to fill the gap toward that movement with technologies that allow consumers to customize their preferences for alternative health care delivery beyond disease management alone [1]. Health apps on mobile phones or tablets have been designed to monitor, assist, and inform patients about individual disease conditions including diabetes, migraines, and asthma [37]. There are only a few specific apps designed to comprehensively manage anticoagulation therapy, although there is a substantial need for ongoing patient education and provider feedback during the course of therapy.

Another form of mHealth technology that is being used in health care is the remote monitoring systems (RMS) or telehealth systems [3]. For example, patients with heart failure living in rural areas or having limited mobility can monitor their blood pressure, heart rate, body weight, and blood oxygen level using peripheral devices (eg, weighting scale blood pressure device, or oximeter) [38]. Such clinical data are transferred to a RMS device that alerts health care providers of clinically meaningful changes in physiological data. Health care providers can then provide patients with immediate feedback based on the data transferred via the RMS system.

Despite the growing use of mHealth technology in health care, many challenges to widespread use remain due to the complexity of health care delivery system in the United States. Health care providers are concerned about the additional workload that the use of mHealth technology may generate, and are unclear about the impact on the provider-patient relationship [1]. Older adults may face more challenges to mHealth technology use due to relatively lower levels of technology proficiency compared to younger populations. However, in this study many of the patients expressed a basic understanding of how mHealth technology works, particularly those related to anticoagulation therapy. Developers of mHealth technology should be aware that older adults can be savvy end users like other age groups, but that technological platforms should be designed with elder-friendly specifications to meet the specific needs of older adults. Elder-friendly suggestions from the participants in this study include simplifying the design of app interfaces, designing bigger screens and keyboards, and providing technical support in using technology aids.

We have been developing a mobile app for older adults on warfarin therapy based on the comments from the participants in this study. The modules of this elder-centered app for warfarin therapy that our research team has developed are shown in the Table 4.

**Table 4:** Modules included in the warfarin therapy app for older adults, mobile app for savvy seniors (MASS), happy and safe life with blood thinners.

Module name	Description
My page	Users enter preferred information that will be useful for their care management. This includes doctor’s information, pharmacy’s information, and emergency contact information. The user for his/her identification can create an icon or picture.
Blood thinners	This educational component provides essential warfarin information that includes medication interactions and food-medication interaction. The information is evidence-based, and written in plain language for easy understanding by users.
Medication	Medication monitoring that creates reminders of dosage and when to take a medication. This schedule can be shared with trusted others such as family, caregivers, or close friends.
Foods	A vitamin K content food list that includes a listing of foods high in vitamin K including ethnic foods.
Body	This module allows users to monitor adverse symptoms such as bruising or bleeding on body. A picture can be taken and saved to the app in order to log the episodes to show a health care provider at an office visit.
Blood test	Monitor and log of the results of blood tests, such as INR level, to track whether they are in the recommended therapeutic range.
Safety tips	Easy and specific safety tips related to warfarin therapy, such as injury prevention and when to call a doctor.
Share	All information logged in the MASS can be shared with trusted other via email. Users are empowered to manage their own health information.
Resources	Additional resources regarding anticoagulation therapy, such as information from federal agencies (eg, Agency for Healthcare Research and Quality, U.S. Department of Agriculture, National Nutrient Databases).

### Health Games for Physical Activities in Older Adults

Games designed to promote exercise (eg, Nintendo Wii, Sony EyeToy, Dance Dance Revolution, or Xbox Kinnet, etc) have been studied in community-dwelling older adults and patients with stroke for rehabilitation [4,39]. Many commercial apps for physical activities that are available in the market are targeted to those who want to lose body weight [40,41]. In addition, there are health apps for diabetes that include a physical activity component along with diet and blood glucose monitoring [42]. Physical activity games can be included in a health app for those on anticoagulation therapy, but designs need to take into account specific safety measures. Older adults on warfarin therapy are at a higher risk for bleeding, and may be limited in engaging in physical activity due to the fear of falling. The participants in this study expressed that they had some experience with commercially available balance games or group games. However, they were concerned about safety issues in regards to the design of the game platforms, and made suggestions for safety strategies to prevent falling while playing games.

### Limitations

There are several limitations to take into consideration. First, the participants were recruited from two anticoagulation clinics affiliated with an academic medical center, and findings may not be generalized outside of the population in this sample due to regional differences in practice. Furthermore, the small sample size and exploratory nature of this qualitative study further limits the generalizability of the findings across all segments of the population. Consequently, the findings from the study are a journalistic representation of the sample’s perceptions and beliefs related to anticoagulation (ie, warfarin), and the potential role of mHealth in promoting self-management. Second, we experienced challenges to recruitment that may have affected the demographics of our sample. Many of the scheduled participants did not show up to the group meetings because of transportation problems or urgent health issues. Therefore, our sample may be more representative of those in better health or those who are more independent. Nevertheless, the findings from this study provide a rich source of data to examine the use of mHealth technology specifically within the context of anticoagulation therapy.

### Conclusions

This study was conducted to generate formative information on the use of mHealth technology among older adults on warfarin therapy, and identify themes that could be captured when designing a valid and reliable instrument based on input from stakeholders. These findings can be used to inform a larger study to design and test mHealth technology in this target population. The next study will include an iterative process to refine the design and effectiveness of the health app in a real life clinical environment. The findings indicate that our older adults on warfarin therapy are interested in mHealth technology specific to warfarin medication management and health games. Further research needs to be done to validate these findings. Elder-friendly designs, technology support, and physical safety using mHealth technology may be useful in this population.

## Multimedia Appendix 1

Patient characteristics.

## Abbreviations

INR: international normalized ratio
IRB: Institutional Review Board
MASS: mobile app for savvy seniors
mHealth: mobile health
NCATS: National Center for Advancing Translational Sciences
NCRR: National Center for Research Resources
NIH: National Institutes of Health
QOL: quality of life
RMS: remote monitoring systems
SMS: short message service
