# Supplementary material for: Attitudes and Preferences on the Use of Mobile Health Technology and Health Games for Self-Management: Interviews With Older Adults on Anticoagulation Therapy
Source: JMIR Mhealth Uhealth. 2014 Jul 23;2(3):e32. doi: 10.2196/mhealth.3196 (PMC4125157; doi:10.2196/mhealth.3196)
Supplement: Supplementary file 1 [file mhealth_v2i3e32_app1.doc]

Appendix 1. Patient characteristics

| Characteristics | | | | | Frequency (N =11) |
| --- | --- | --- | --- | --- | --- |
| **Demographic** | | | | |  |
| Age (years) | | | | |  |
|  | | | Mean (SD) | | 74.9 (7.5) |
|  | | | Median | | 75 |
|  | | | Range | | 61-89 |
| Gender (male) | | | | | 8 |
| Ethnicity/race | | | | |  |
|  | | | Caucasian | | 6 |
|  | | | Hispanic | | 3 |
|  | | | Asian | | 2 |
| Education, high school completion or above | | | | | 8 |
| Living situation, living with family (vs living alone) | | | | | 10 |
| Currently employed | | | | | 2 |
| Insured | | | | | 10 |
| Having a primary care provider | | | | | 9 |
| **Comorbidities** | | | | |  |
|  | | | Stroke | | 4 |
|  | | | Diabetes | | 3 |
|  | | | Hypertension | | 3 |
|  | | | Arthritis | | 3 |
|  | | | | Heart failure | 2 |
|  | | | | Atrial fibrillation | 1 |
|  | | | | Lupus | 1 |
|  | | | | Multiple comorbidity (≥1) | 5 |
| **Years taking blood thinner (warfarin)** | | | | |  |
|  | | | | Mean (SD) | 5.45 (4.8) |
|  | | | | Median | 5 |
|  | | | | Range | 3 weeks-16 years |
| Have ever skipped taking blood thinner? (yes) | | | | | 6 |
|  | | | | |  |
| **Technology related** | | | | |  |
|  | | | | |  |
| Have a home computer | | | | | 6 |
| Use a computer at all including at home, library, community center, other places | | | | | 6 |
| Reasons for computer use | | | | |  |
|  | | Internet and email | | | 6 |
|  | | Word processing | | | 4 |
|  | | Scheduling (calendar or reminder) | | | 2 |
|  | | Managing household finances | | | 0 |
|  | | Photos or music | | | 0 |
|  | | Playing games | | | 0 |
|  | | Other | | | 1 |
| Frequency of computer use | | | | |  |
|  | Never | | | | 5 |
|  | Once per month or less | | | | 0 |
|  | 2-3 times per month | | | | 0 |
|  | Every week | | | | 0 |
|  | Every day | | | | 5 |
| Have ever used a smart phone/tablet for health apps such as medication reminders or weight management (yes) | | | | | 2 |
| Frequency of health apps use | | | | |  |
|  | Once per month or less | | | | 2 |
| Have ever played computer/videogames (yes) | | | | | 5 |
|  | Computer games | | | | 3 |
|  | Videogames (eg, Wii, PlayStation, Xbox, etc) | | | | 2 |
|  | Games on phones/tablets | | | | 1 |
| Frequency of game playing | | | | |  |
|  | Never | | | | 10 |
|  | Once per month or less | | | | 1 |
